# A modified transosseous quadriceps tendon refixation technique demonstrates advantageous primary stability: A biomechanical investigation

**DOI:** 10.1002/jeo2.70695

**Published:** 2026-03-24

**Authors:** Nora S. Michel, Lucas K. Palma Kries, Andre Frank, Michael J. Raschke, J. Christoph Katthagen, Simon Oeckenpöhler, Christoph Kittl, Adrian Deichsel, Philipp A. Michel

**Affiliations:** ^1^ Department of Trauma, Hand and Reconstructive Surgery University Hospital Münster Münster Germany; ^2^ Faculty of Engineering and Computer Science Osnabrück University of Applied Sciences Osnabrück Germany

**Keywords:** primary stability, quadriceps tendon repair, suture anchors, transosseous sutures, transpatellar refixation

## Abstract

**Purpose:**

Recent biomechanical research suggests suture anchor (SA) refixation may be superior to conventional transosseous longitudinal (TL) repair for quadriceps tendon ruptures. This study aimed to evaluate whether a modified transosseous technique with anteriorly angled drill holes enhances primary stability compared to the aforementioned techniques.

**Methods:**

Twenty‐one human cadaveric patellae were randomly assigned to three groups (*n* = 7): SA (two SAs), TL (three longitudinal transosseous drill holes) and TA (three anteriorly angled 45° transosseous drill holes). Identical suture material (Ethibond #2) and a four‐strand suture configuration were used in all groups. Prior to fixation, computed tomography (CT) scans measured bone mineral density (BMD) and cortical thickness. Biomechanical testing simulated early postoperative loading using a universal testing machine: preload (20 N for 30 s), cyclic loading (500 cycles, 20–100 N at 1 Hz) and a load‐to‐failure test (20 mm/s). As primary outcome, ultimate load to failure was recorded; secondarily, cyclic elongation and stiffness were measured. One‐way analysis of variance (ANOVA) with Bonferroni‐corrected post hoc *t* tests was used for statistical analysis.

**Results:**

The TA group had a significantly higher ultimate load to failure than the TL group (472.7 ± 22.4 vs. 344.8 ± 108.8 N, *p* < 0.05). No significant differences were found between the SA group (438.9 ± 77.4 N) and the other groups. The TL group showed significantly greater cyclic elongation (6.22 ± 1.03 mm) compared to the SA group (4.42 ± 0.82 mm, *p* < 0.01). The TA group (5.03 ± 0.73 mm) showed no significant difference compared to either group. Stiffness did not differ significantly (*p* > 0.05).

**Conclusion:**

The modified transosseous technique with anterior drill holes yields higher failure loads and less elongation than the conventional TL method, with biomechanical performance comparable to SA fixation, supporting its use as a viable alternative.

**Level of Evidence:**

N/A.

**Abbreviations**
ANOVA: analysis of variance
BMD: bone mineral density
CT: computed tomography
IRB: institutional review board
QCT: quantitative computed tomography
SA: suture anchor
SD: standard deviation
TA: transosseous anterior
TL: transosseous longitudinal

## INTRODUCTION

Complete quadriceps tendon ruptures typically require surgical intervention, with osseous refixation of the tendon to the patella considered the current gold standard [14, 20]. Although clinical outcomes are generally favourable, major complications, including re‐rupture and persistent strength deficits, are reported in up to 30% of patients [2, 7, 10, 17, 22]. Established techniques for osseous refixation include transosseous drill holes oriented longitudinally through the patella and the use of suture anchors placed at the proximal patellar pole.

Several biomechanical studies and systematic reviews suggest that suture anchor (SA) refixation may offer superior biomechanical properties and potentially reduced access‐related morbidity compared to conventional transosseous repair using longitudinal drilling [12, 16, 24, 25]. However, SA fixation is associated with certain disadvantages, such as an increased volume of implanted foreign material and higher implant‐related costs [1].

This study introduces and evaluates a modified transosseous refixation technique. In contrast to conventional longitudinal drilling, this technique involves creating drill holes at a 45° angle relative to the longitudinal axis of the patella, exiting on the anterior patellar surface. This approach aims to create a robust bone bridge within the thicker anterior cortical bone for suture passage.

The purpose of this biomechanical investigation was to evaluate the primary stability of this modified transosseous technique. It was hypothesised that the modified transosseous anterior (TA) refixation would exhibit higher ultimate load to failure compared to the conventional transosseous longitudinal (TL) refixation and SA, thus representing a viable alternative for quadriceps tendon repair. Additionally, the cyclic elongation and stiffness of the tested refixation techniques will be compared.

## MATERIALS AND METHODS

An a‐priori power analysis was conducted using G*Power (Version 3.1.9.7; Heinrich‐Heine‐Universität Düsseldorf) [8, 9] to determine the minimum required sample size. Based on data from a previous study by Michel et al. [21] regarding load to failure (assuming a mean difference of 150 N and a standard deviation (SD) of 82 N, yielding an effect size *f* ≈ 0.9), a sample size of *n* = 7 per group was calculated to achieve a power of >80% (actual power ≈ 0.85) at a significance level of *α* = 0.05 for detecting a significant difference between groups using analysis of variance (ANOVA).

A total of 21 human cadaveric patellae were obtained from ‘Institute of Anatomy in Lübeck’. These specimens were procured from knee joints with no documented history of prior injury, surgery or osteoarthritis. The cohort comprised 15 female and 6 male donors, with a mean age of 80.2 ± 7.5 years (range: 62–91 years). Each specimen was visually inspected to exclude any gross anatomical abnormalities or pre‐existing damage before testing.

Ethical approval for this study was granted by the institutional review board (IRB) of the University of Münster (IRB reference number: AZ 2014‐421‐f‐N).

Specimens were stored frozen at −20°C in double vacuum‐sealed packaging and thawed at room temperature for 24 h before preparation and testing. The patellae were meticulously dissected from the surrounding soft tissues, including the quadriceps and patellar tendons, and then prepared according to their randomly allocated experimental group.

The 21 specimens were matched for age and sex and then randomly assigned to one of three experimental groups (*n* = 7 per group):

- Group SA (suture anchor fixation): Refixation using two Mitek Fastin RC 6.5 mm suture anchors (DePuy Synthes).
- Group TL (conventional transosseous longitudinal fixation): Refixation using three longitudinally oriented drill holes.
- Group TA (modified transosseous anterior fixation): Refixation using three drill holes angled 45° anteriorly.

### Surgical technique

All simulated repairs utilized a standardized four‐strand construct of Ethibond® #2 non‐absorbable braided polyester suture material (Ethicon).

- Group SA: Two 2.5 mm pilot holes were drilled longitudinally into the proximal patellar pole using a drill guide, positioned at the junction of the medial/central and central/lateral thirds of the proximal patellar pole. A Mitek Fastin RC 6.5 mm suture anchor, double‐loaded with Ethibond® #2 suture, was inserted into each pilot hole. This resulted in four suture strands spanning the intended repair site (Figures 1a and 2a).
- Group TL: Three 2.5 mm drill holes were created using a drill guide in the longitudinal (superior‐inferior) direction. These drill holes formed three bone tunnels that divided the proximal patellar pole into four equal segments. Suture passage followed the technique described by Kindya et al. [15], whereby two suture strands were passed through each of the two lateral tunnels at the proximal patellar pole and then directed superiorly to exit through the central tunnel at the distal patellar pole, yielding four suture strands for fixation (Figures 1b, 2b and 3).
- Group TA: Three 2.5 mm drill holes were created using a drill guide, similarly dividing the proximal patellar pole into four equal segments. These drill holes were directed from the proximal patellar surface to form tunnels exiting on the anterior (ventral) patellar surface approximately at a 45° angle relative to the longitudinal axis of the patella. A four‐strand suture configuration, analogous to the TL group, was employed. For each of the two lateral tunnels, two suture strands were passed from the proximal surface to exit on the anterior surface using a curved suture passer (DS‐43; Fa. Serag‐Wiessner). All four strands were then passed superiorly through the central tunnel, which also exited on the anterior surface, thereby creating a transosseous bone bridge for suture fixation (Figures 1c, 2c and 3).

**Figure 1:**
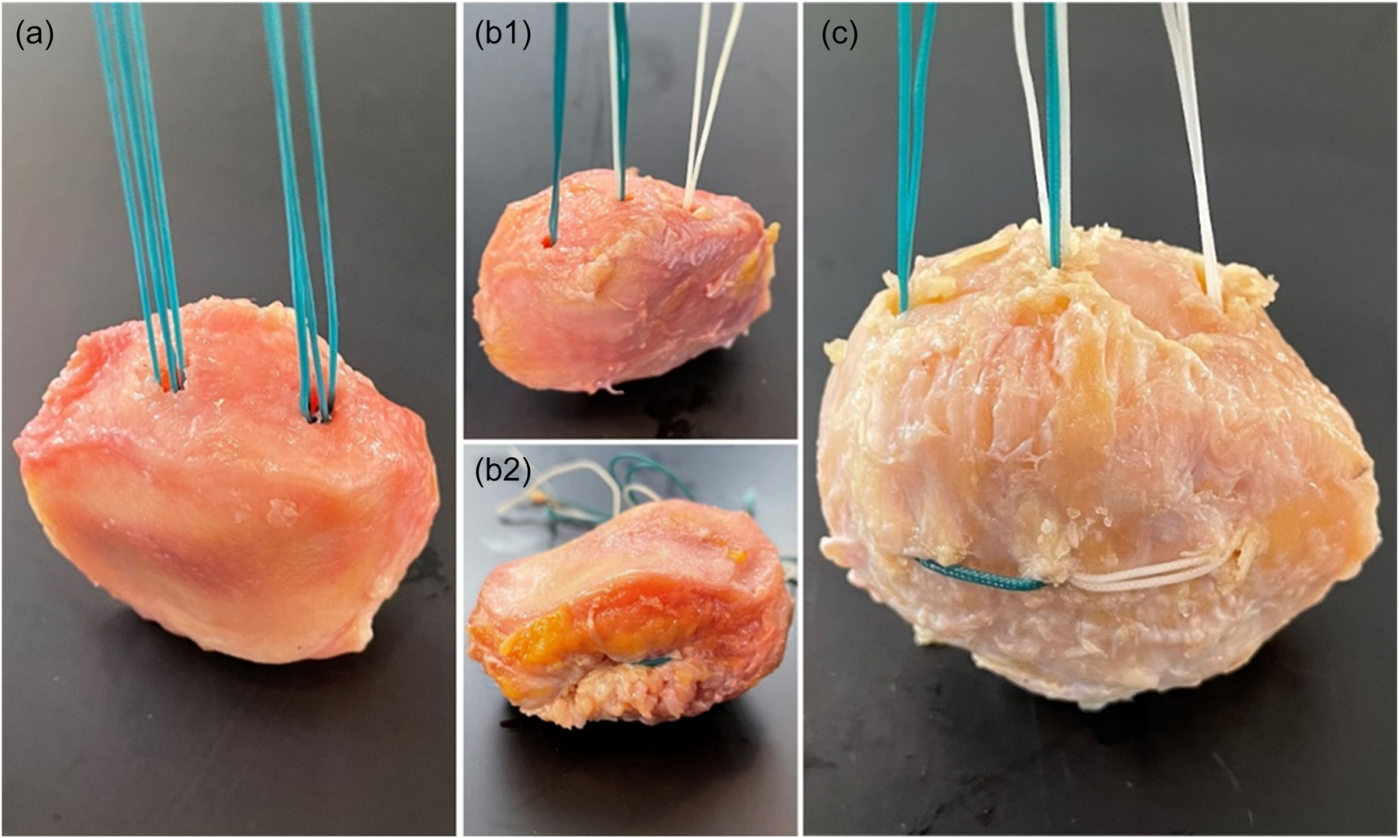
Schematic representation of the three surgical preparation techniques using human patellae. All repairs utilized a four‐strand Ethibond #2 suture construct. (a) Suture anchor (SA) technique: Two Mitek Fastin RC 6.5 mm anchors implanted in the proximal patellar pole. (b1) Conventional transosseous longitudinal (TL) technique: Three drill holes oriented longitudinally, with sutures exiting at the distal patellar pole. (b2) Distal view of suture exit points in the TL technique. (c) Modified transosseous anterior (TA) technique: Ventral view of a patella illustrating three drill channels exiting on the anterior patellar surface at a 45° angle.

**Figure 2:**
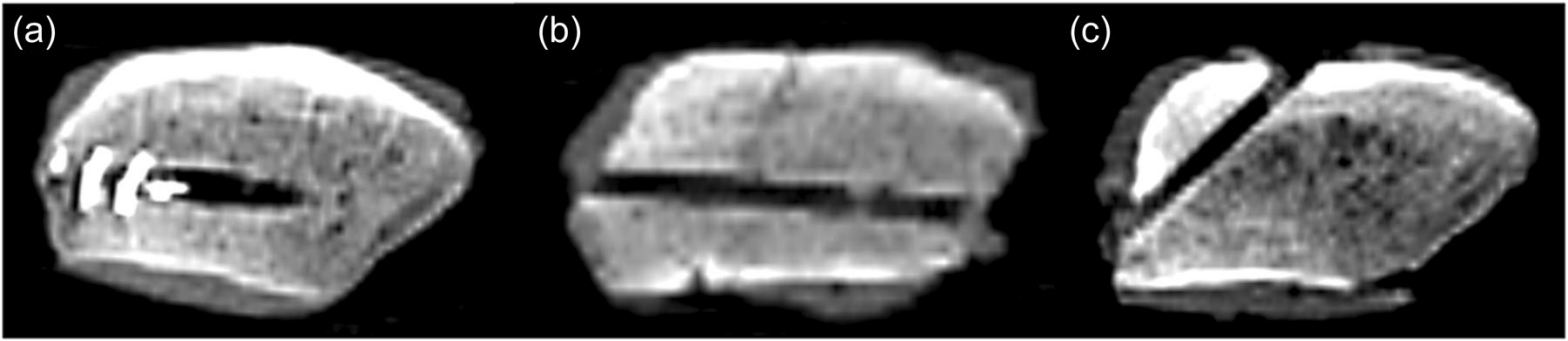
Sagittal CT sectional image planes illustrating the drill channel trajectories and fixation constructs for each technique. (a) Suture anchor (SA). (b) Transosseous longitudinal (TL). (c) Transosseous anterior (TA). CT, computed tomography.

**Figure 3:**
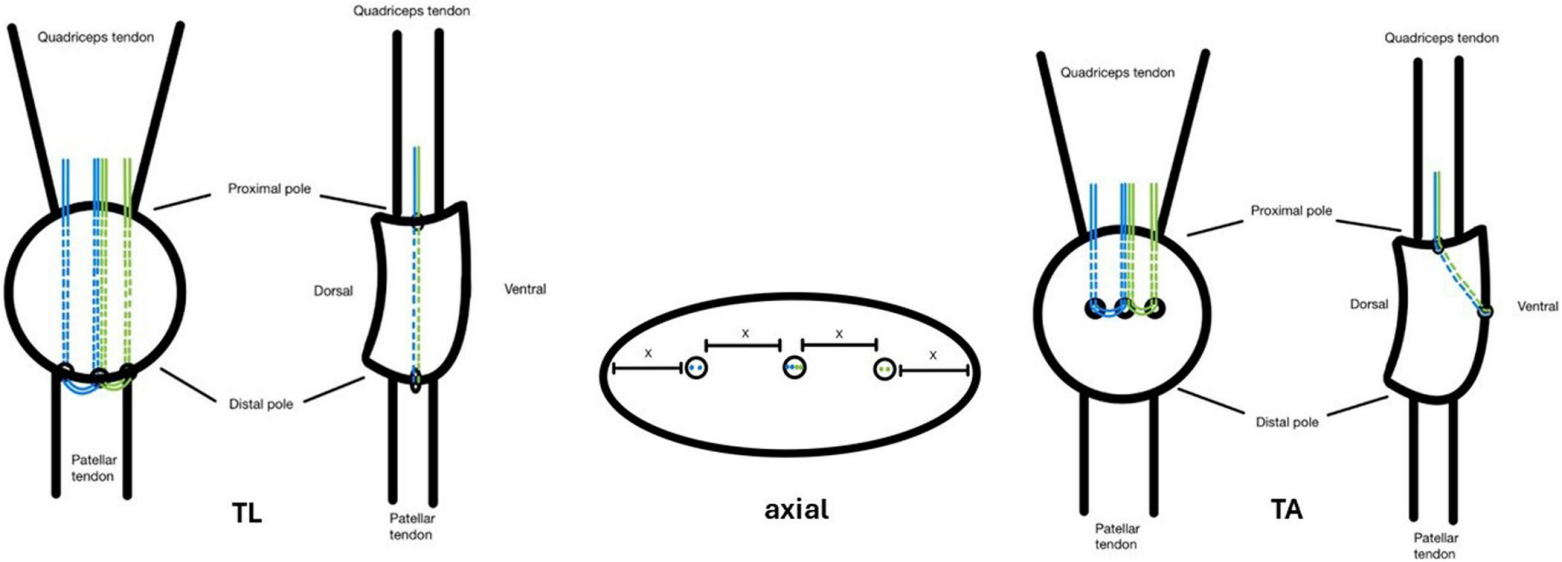
Schematic illustration of the TL (left) and TA technique (right) in the coronal and sagittal planes. For the TL technique, three drill channels were created in the longitudinal direction from the proximal patella pole to the distal patella pole, and two sutures were passed through each of the lateral drill channels. For the TA technique, three drill channels were created approximately at a 45° angle from the proximal patellar pole to the ventral side of the patella, and two sutures were passed through each of the lateral drill channels. All four sutures were passed back to the proximal patella pole through the middle drill channel. The drill channels divided the proximal patella pole into four equal mediolateral sections (axial view). The distance *x* depends on the mediolateral size of the proximal patella pole. In the present study, *x* was 7–9 mm in the different specimens. TA, transosseous anterior; TL, transosseous longitudinal.

### Biomechanical testing

Biomechanical evaluation of the prepared patellar constructs was performed using a universal testing machine (Z005‐TN2A; Zwick/Roell), equipped with a 5 kN load cell. Each construct underwent a sequence of preconditioning, cyclic tensile testing, and finally, a load‐to‐failure test. The testing machine was controlled, and data were acquired using TestXpert III software (Zwick/Roell). Force (N) and elongation (mm) were recorded continuously at a sampling rate of 100 Hz.

The patella was rigidly fixed in a custom‐designed cylindrical clamp using eight radially oriented set steel screws (M5, 30 mm), a method adapted from previous studies to ensure stability and prevent slippage during testing [24, 25] (Figure 4). The screws were uniformly tightened with 5 Nm using a torque wrench.

**Figure 4:**
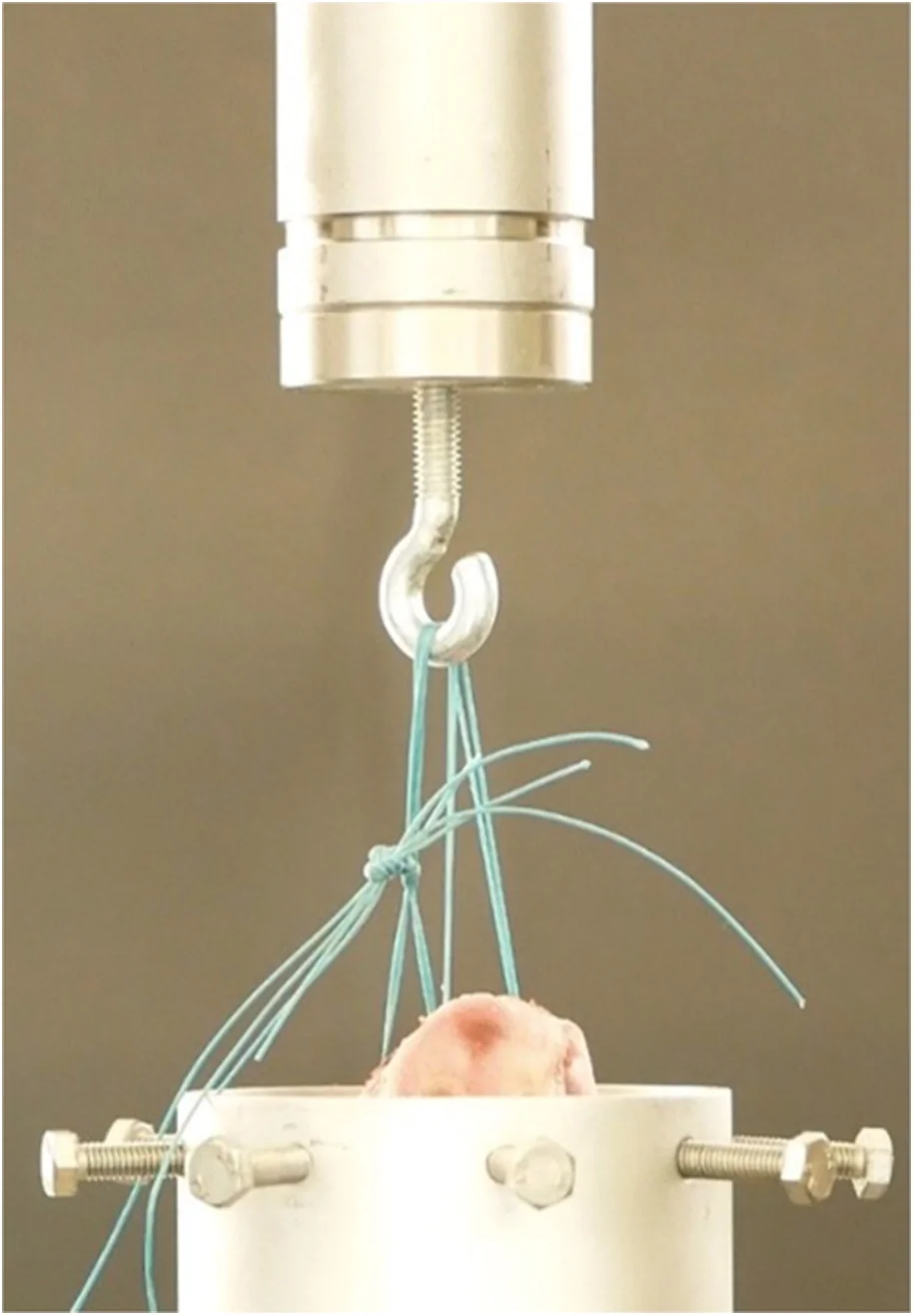
Experimental setup showing a human patella (exemplified by the TL group) rigidly clamped in the custom‐designed fixture within the universal testing machine (Zwick/Roell). TL, transosseous longitudinal.

A stainless‐steel hook, attached to the actuator of the testing machine, served to transmit the tensile force to the suture construct. The four suture strands from each repaired specimen were securely tied to this hook using a standardized fisherman's knot with eight counter‐directional throws. This knot configuration was chosen for its high tensile strength and resistance to slippage, aiming to ensure that failure occurred at the bone‐suture interface rather than at the knot.

The test protocol was designed to simulate the forces experienced during early postoperative rehabilitation.

1. Preconditioning: Constructs were preconditioned with a static tensile preload of 20 N for 30 s.
2. Cyclic loading: Subsequently, constructs underwent 500 cycles of sinusoidal tensile loading between 20 and 100 N at a frequency of 1 Hz. This regimen was based on established protocols for simulating physiological loads during early passive range of motion exercises [21]. Total elongation of the construct was defined as the displacement measured after the completion of the 500th cycle relative to the position after preconditioning.
3. Load‐to‐failure test: Finally, a destructive load‐to‐failure test was performed by applying tensile load at a constant displacement rate of 20 mm/s until construct failure. Ultimate load to failure (N) was defined as the peak force recorded during this test. Stiffness (N/mm) was calculated from the linear region of the load‐displacement curve generated during the load‐to‐failure test. Visual observation was used to determine where the failure occurred in the construct.

### Statistical analysis

Statistical analyses were performed using GraphPad Prism (Version 8.0.0; GraphPad Software). All data are presented as mean ± SD. Normality of data distribution was assessed using the Shapiro–Wilk test.

Biomechanical parameters (cyclic elongation, ultimate load to failure, stiffness) and computed tomography (CT)‐derived bone mineral density (BMD) values for the three experimental groups were compared using one‐way ANOVA. If the ANOVA indicated a significant overall difference, post hoc pairwise comparisons were performed using *t* tests with Bonferroni correction for multiple comparisons.

An independent samples *t* test was used to compare the mean cortical thickness of the ventral patella with that of the distal patellar pole.

Statistical significance was set at *p* < 0.05 for all analyses.

## RESULTS

The 21 tested specimens could be evaluated without complications. Mean age of the body donors was 79.5 ± 9.1 years with a minimum age of 62 years and a maximum age of 94 years. Of the tested knees, 15 (71%) were female and 6 (29%) were male.

### Cyclic elongation

After 500 cycles of loading, the TL group (6.22 ± 1.03 mm) exhibited significantly greater elongation compared to the SA group (4.42 ± 0.82 mm) (*p* = 0.0012). The TA group (5.03 ± 0.73 mm) showed no significant difference in cyclic elongation when compared to the SA group (*p* = 0.47) or the TL group (*p* = 0.06) (Figure 5, Table 1).

**Figure 5:**
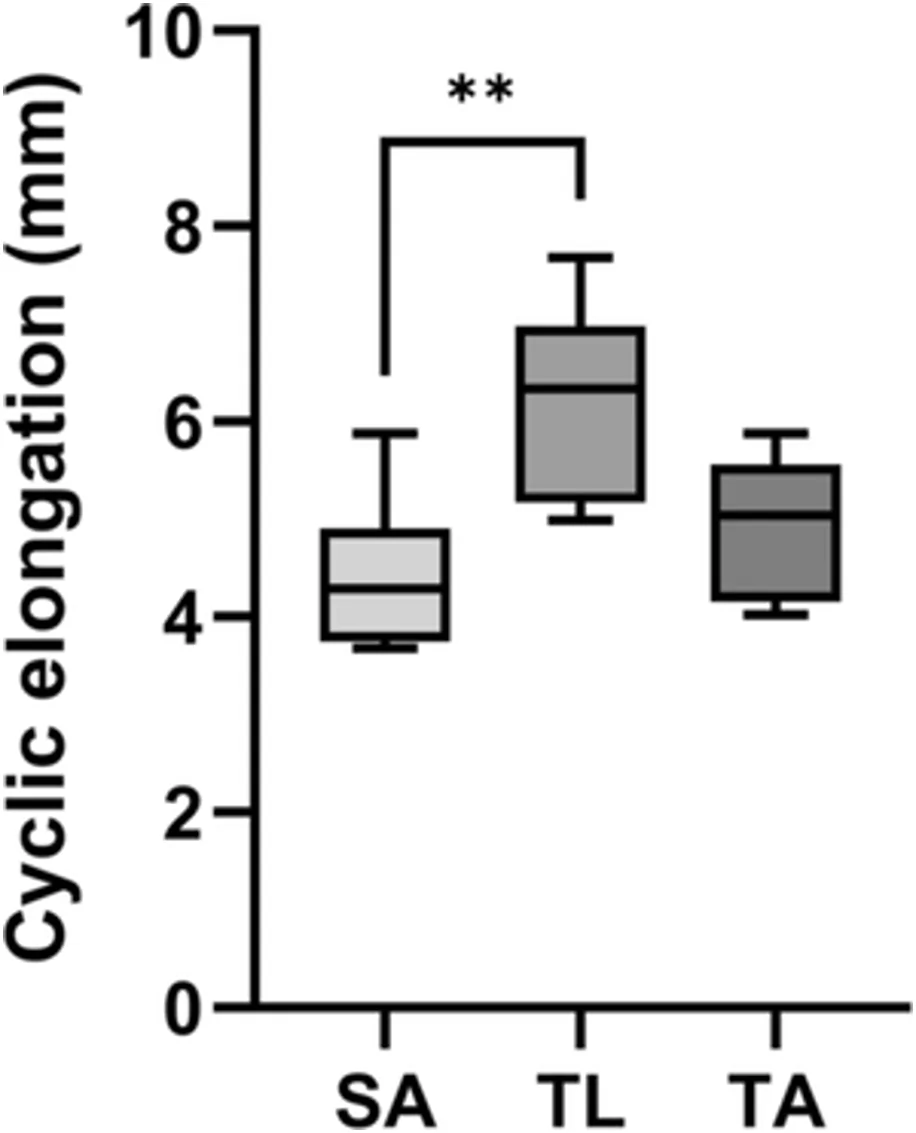
Boxplot illustrating cyclic elongation (mm) of the constructs in the SA, TL and TA groups after 500 cycles of loading. ** indicates *p* < 0.01. Whiskers represent minimum and maximum values. SA, suture anchor; TA, transosseous anterior; TL, transosseous longitudinal.

**Table 1:** Cyclic elongation, load to failure and stiffness of the different groups and comparison between groups.

Parameter	SA (*n* = 7)	TL (*n* = 7)	TA (*n* = 7)	SA vs. TL (*p*)	SA vs. TA (*p*)	TL vs. TA (*p*)
Cyclic elongation (mm)	4.42 ± 0.82 (3.68–5.88)	6.22 ± 1.03 (4.99–7.68)	5.03 ± 0.73 (4.02–5.88)	0.006	n.s.	n.s.
Load to failure (N)	438.9 ± 77.4 (278.6–497.5)	344.8 ± 108.8 (163.0–480.9)	472.7 ± 22.41 (424.3–498.9)	n.s.	n.s.	0.01
Stiffness (N/mm)	31.23 ± 7.88 (15.52–37.10)	23.62 ± 6.83 (17.15–36.66)	28.28 ± 3.07 (22.76–31.71)	n.s.	n.s.	n.s.

*Note*: Data are reported as mean ± standard deviation and range (minimum − maximum).

Abbreviations: mm, millimetre; N, Newton; n.s., not significant; SA, suture anchor; TA, transosseous anterior; TL, transosseous longitudinal.

### Load to failure

The ultimate load to failure was highest in the TA group (472.7 ± 22.41 N). The TL group (344.8 ± 108.8 N) demonstrated a significantly lower ultimate load to failure compared to the TA group (*p* = 0.013). No significant difference in ultimate load to failure was observed between the SA group (438.9 ± 77.4 N) and the TA group (*p* = 0.69), nor between the SA group and the TL group (*p* = 0.11) (Figure 6, Table 1).

**Figure 6:**
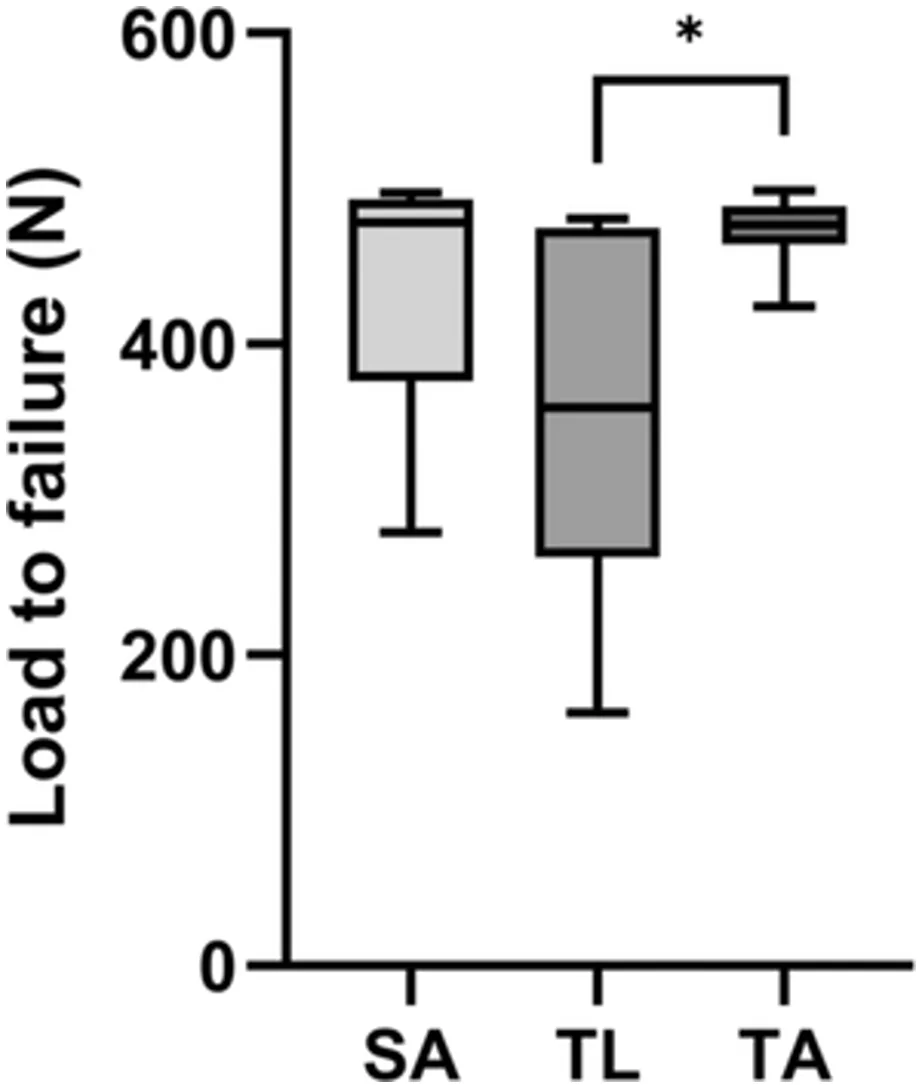
Boxplot illustrating ultimate load to failure (N) of the constructs in the SA, TL and TA groups. * indicates *p* < 0.05. Whiskers represent minimum and maximum values. SA, suture anchor; TA, transosseous anterior; TL, transosseous longitudinal.

### Mechanism of failure

In the SA group, the construct failed in four cases due to a suture tear and in three cases due to the suture anchor tearing out of the bone. In the TL group, in six constructs, failure occurred due to suture tears next to the exit at the distal drill hole, and in one case, because of the suture cutting out from the bone. In the TA group, the constructs failed in all seven tests due to a suture tear. No displacement of the patella from the custom‐made clamp was observed.

### Stiffness

No significant differences in construct stiffness were found among the SA (31.23 ± 7.88 N/mm), TL (23.62 ± 6.83 N/mm) and TA (28.28 ± 3.07 N/mm) groups (*p* > 0.05 for all pairwise comparisons) (Figure 7, Table 1).

**Figure 7:**
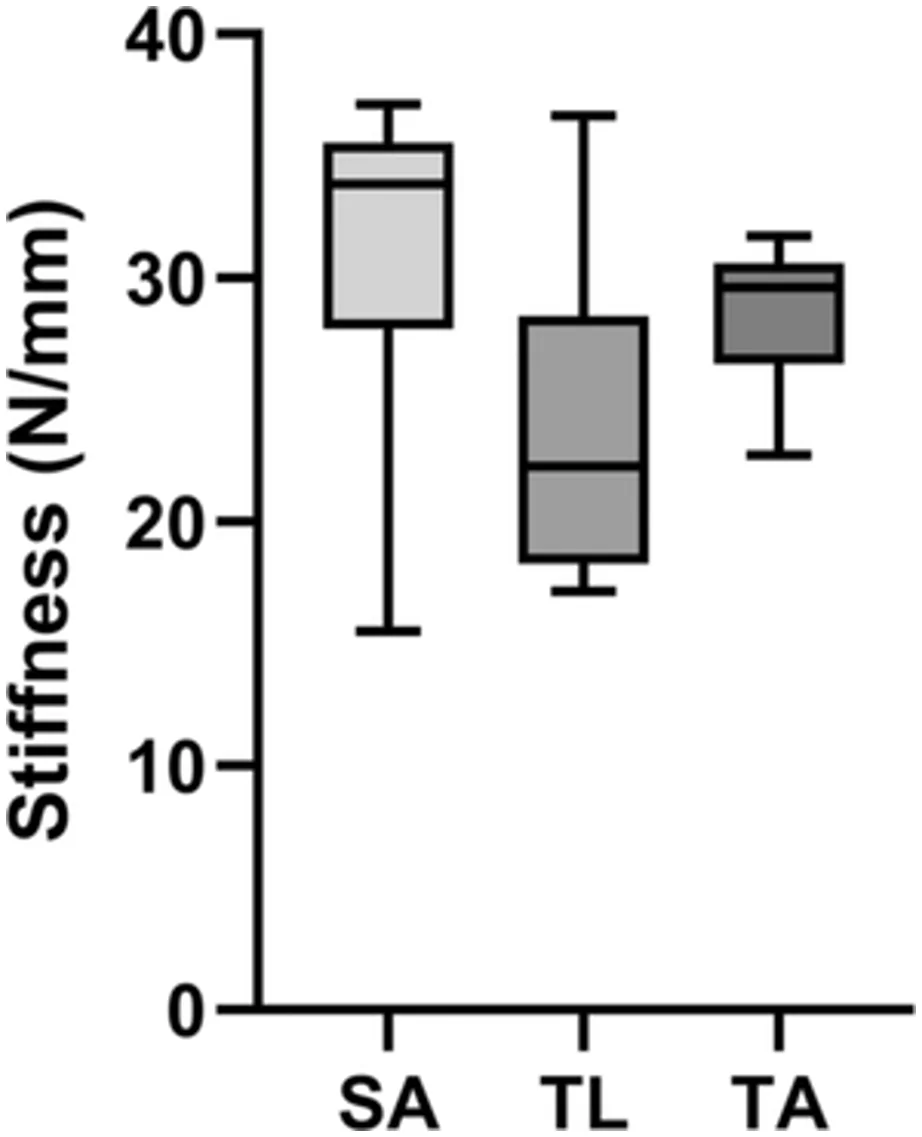
Boxplot illustrating construct stiffness (N/mm) of the SA, TL and TA groups. Whiskers represent minimum and maximum values. SA, suture anchor; TA, transosseous anterior; TL, transosseous longitudinal.

## DISCUSSION

The principal finding of this biomechanical study is that the modified transosseous quadriceps tendon refixation technique, utilizing drill tunnels exiting on the ventral patellar surface (TA group), demonstrated a significantly higher ultimate load to failure and a trend towards less cyclic elongation compared to the conventional longitudinal transpatellar technique (TL group). Furthermore, the biomechanical performance of this novel TA technique, in terms of both cyclic elongation and ultimate load to failure, was comparable to that of suture anchor fixation (SA group).

Clinical studies comparing quadriceps tendon refixation using suture anchors or transosseous techniques with longitudinal drilling do not show significant differences in functional outcome scores, while the range of motion is slightly increased postoperatively using the transosseous refixation technique [20]. Since existing biomechanical studies favour suture anchors over the use of transosseous refixation techniques [5], the presented technique with transosseous refixation using anterior drilling could show comparable biomechanical stability to the use of suture anchors. While clinical outcomes of the presented techniques still need to be investigated, it could be a viable alternative to the use of suture anchors.

These findings support our primary hypothesis that redirecting transosseous tunnels to the thicker anterior patellar cortex enhances primary fixation stability. The significantly greater cortical thickness on the ventral aspect of the patella compared to its distal pole, as confirmed by our CT analysis, likely provides a more robust bone stock for suture purchase, thereby contributing to the increased failure loads observed in the TA group. Additionally, the less acute angle of suture exit from the anteriorly placed tunnels might distribute stress more favourably along the suture strands and at the bone‐suture interface compared to the sharper angle of the suture strands exiting from the distal drill holes in the TL technique, thus potentially reducing the risk of suture cut‐through or bone bridge fracture under load. This is supported by the observed mechanism of failure. In the TL group, the suture tears occurred next to the exit of the distal drill holes, and in one case, suture cutout was observed, while in the TA group, suture tears occurred proximal to the patella. This observation can only indicate a trend, which, due to the limited sample size, cannot be considered conclusive.

Our results align with the evolving understanding of optimal fixation strategies in orthopaedic surgery, where leveraging areas of superior bone quality is paramount [19]. While previous biomechanical studies comparing suture anchors to conventional transosseous tunnels for quadriceps tendon repair [11, 12, 15, 16, 24, 25] have yielded somewhat mixed results, a systematic review by Dankert et al. [5] suggested potential advantages for suture anchors regarding gap formation and ultimate failure load, though not always reaching statistical significance. It is crucial to note, as highlighted by the authors, that heterogeneity among studies, including variations in testing protocols, used implants and specimen characteristics, can influence meta‐analytic conclusions. Our study confirms the trend observed in biomechanical studies, showing significantly greater cyclic elongation in the TL group than in the SA group [5]. Also, the ultimate load to failure was smaller in the TL group than in the SA group, although this difference was not statistically significant. This is consistent with the findings of previous biomechanical research [5]. However, to our knowledge, the TA technique is not described in the existing literature, so there is no data on the biomechanical properties of this procedure. The suture anchors used in this study are commonly used for quadriceps tendon refixation [4] and show no significant differences regarding load to failure and intraosseous displacement compared to other cancellous‐type suture anchors [18].

One limitation of many prior studies was the absence of detailed bone quality assessment or rigorous matching procedures, which could introduce bias. A strength of the current investigation is the inclusion of CT‐based BMD and cortical thickness measurements (see Supporting Information S1: Data 1), confirming comparable baseline bone quality across groups, and the matching of specimens for age and sex, thereby minimizing these potential confounders.

The clinical implications of our findings are noteworthy. The TA technique offers a biomechanically sound alternative to both conventional transosseous repair and suture anchor fixation. Compared to the TL technique, the TA technique provides superior primary stability. Compared to suture anchors, the TA technique achieves comparable biomechanical performance while potentially offering advantages such as reduced implant costs [13, 26] and a decreased volume of foreign material left in situ, which may be relevant in discussions about long‐term implant‐related complications or revision scenarios. Furthermore, when using the TA technique, it is sufficient to access only the ventral side of the patella. Exposing the distal part of the patella, as required for the TL technique, may involve partial patellar tendon detachment, resulting in greater soft tissue damage. The presented TA technique does not require exposure of the distal patellar pole, potentially reducing surgical morbidity. However, a potential concern with anteriorly exiting sutures is the possibility of implant‐related irritation or prominence, particularly during activities involving kneeling. This problem could possibly be assessed through coverage of the ventral sutures by reconstructing the fibres of the extensor apparatus running ventral to the patella. In addition, the weakening of the tension side of the patella by the three ventral drilling holes could potentially result in a patellar coronal fracture. However, no such fractures were observed in this investigation, despite the fact that the cadaveric cohort was older and more feminine than the typical patient with a quadriceps tendon tear [3]. Nevertheless, these aspects warrant careful consideration and evaluation in future clinical studies.

The limitations of this study should be acknowledged. Firstly, this was an in vitro biomechanical study evaluating the bone‐suture interface in isolation, without incorporating a biological tendon graft. Therefore, we measured construct elongation and failure at the fixation site, not true gap formation at a tendon‐patella interface. However, by standardizing the suture material and configuration across all groups, we aimed to isolate the effect of the bone fixation technique. This simplified model also enhances reproducibility by eliminating the inherent variability associated with cadaveric tendon tissue quality and suture‐tendon interaction. Secondly, the choice of Ethibond #2 suture, while consistent across groups, may not represent the highest‐strength suture material currently available. Other materials, such as high‐strength non‐absorbable sutures (e.g., FiberWire), have demonstrated superior mechanical properties [23] and might yield different absolute failure loads. Nevertheless, the relative performance differences observed between the fixation techniques in this study are likely to be maintained. Thirdly, the demographic profile of our cadaveric cohort (predominantly elderly females) may not perfectly reflect the typical patient population sustaining acute quadriceps tendon ruptures, which often includes younger, more active individuals, particularly males [3]. While bone quality was assessed and found to be comparable between groups, the generalizability of our findings to populations with different bone densities or activity levels requires further investigation. Finally, while we used established titanium suture anchors, the biomechanical performance of other anchor types (e.g., all‐suture anchors, knotless anchors) might differ [6], although current evidence does not point to a universally superior anchor design for this specific application.

In this study, cyclic testing was performed with 100 N, which does not simulate a dynamic physiological muscle force. The aim of this study was to simulate passive motion during the early phases of rehabilitation and used an established testing protocol [21].

Also, as this was a time zero study, long‐term healing or creep of the refixation during a the rehabilitation cannot be assessed and further studies are needed for the evaluation of these parameters.

## CONCLUSION

This biomechanical study demonstrates that a modified transosseous quadriceps tendon refixation technique, involving drill holes directed to exit on the anterior patellar surface, provides significantly higher ultimate load to failure and comparable cyclic elongation relative to conventional longitudinal transosseous tunnels. The biomechanical performance of this modified technique is comparable to that of suture anchor fixation. The enhanced stability afforded by the anterior tunnel placement is likely attributable to the engagement of the thicker ventral patellar cortex. These findings suggest that the modified TA technique is a biomechanically robust and potentially cost‐effective alternative for quadriceps tendon repair, worthy of further clinical investigation.

## AUTHOR CONTRIBUTIONS

**Nora S. Michel**: Conceptualization; methodology; investigation; data curation; writing—original draft. **Lucas K. Palma Kries**: Conceptualization; methodology; formal analysis; writing—original draft; writing—review and editing. **Andre Frank**: Investigation; data curation. **Michael J. Raschke**: Supervision; writing—review and editing. **J. Christoph Katthagen**: Supervision; writing—review and editing. **Simon Oeckenpöhler**: Conceptualization; methodology; supervision; writing—review and editing. **Christoph Kittl**: Supervision; writing—review and editing. **Adrian Deichsel**: Writing—review and editing. **Philipp A. Michel**: Conceptualization; methodology; investigation; formal analysis; supervision; writing—original draft; writing—review and editing.

## CONFLICT OF INTEREST STATEMENT

The authors declare no conflicts of interest.

## ETHICS STATEMENT

The specimens were dissected and biomechanically tested under the approval of the Institutional Ethics Committee of the University of Muenster (file number AZ 2014‐421‐f‐N).

## Supporting information

Supplementary Materials QT Refixation.

## Data Availability

The data that support the findings of this study are available from the corresponding author upon reasonable request.
