# Supplementary material for: A modified transosseous quadriceps tendon refixation technique demonstrates advantageous primary stability: A biomechanical investigation
Source: J Exp Orthop. 2026 Mar 24;13(1):e70695. doi: 10.1002/jeo2.70695 (PMC13679893; doi:10.1002/jeo2.70695)
Supplement: Supplementary file 1 [file JEO2-13-e70695-s001.docx]

**Supplementary materials**

Bone Density and Cortical Thickness Measurement

Quantitative computed tomography (QCT) scans (Siemens Somatom Definition AS, Siemens Healthineers, Erlangen, Germany) were performed on all patellae prior to surgical preparation to assess bone quality. RadiAnt DICOM Viewer software (Version 2020.2, Medixant, Poznan, Poland) was used for measuring bone mineral density (BMD) and cortical thickness.

For each specimen, three axial and three sagittal scan planes were defined. Ventral cortical thickness was measured at three distinct locations (lateral, central, and medial) on each of the three axial planes at locations on the ventral side of the patella in an area where the drilling holes are planned to be created in the TA group. Similarly, distal cortical thickness (at the patellar apex) was measured at three locations on each of the three sagittal planes at the distal end of the patella in an area where the longitudinal drill holes were planned to be positioned (Fig. S1).

**Figure S1:** Representative computed tomography (CT) images demonstrating cortical thickness measurement sites.
(A) Axial image plane: Visualization of ventral cortical bone (highlighted in light blue). Yellow crosses indicate three exemplary measurement points for ventral cortical thickness (lateral, central, medial). (B) Sagittal image plane: Visualization of distal cortical bone at the distal patellar pole (highlighted in light blue). Yellow crosses indicate three exemplary measurement points for distal cortical thickness.

BMD of the cancellous bone was measured within standardized regions of interest (ROIs) on each scan plane. Hounsfield Units (HU) obtained from these ROIs were converted to volumetric BMD (mg/cm³) using an established calibration method based on a phantom of known densities [1]. The multiple measurements for cortical thickness and BMD for each specimen were then averaged to yield a single value per parameter per specimen.

Computer tomography scans

Across all specimens, the mean ventral cortical thickness (2.92 ± 0.58 mm) was significantly greater than the mean cortical thickness at the distal patellar pole (0.97 ± 0.11 mm) (P < 0.0001) (Fig. S2, Tab. S1).

**Figure S2:** Boxplot comparing mean cortical thickness (mm) of the ventral patellar region versus the distal patellar pole. **** indicates P < 0.0001. Whiskers represent minimum and maximum values.

There were no significant differences in CT-derived mean BMD values among the SA (210.8 ± 51.9 mg/cm³), TL (229.0 ± 31.2 mg/cm³), and TA (212.9 ± 51.0 mg/cm³) groups (P = 0.68), indicating comparable baseline bone quality across the experimental groups (Tab. S2).

|  | **Ventral cortex (n = 21)** | **Distal cortex (n = 21)** | **P** |
| --- | --- | --- | --- |
| **Cortical thickness (mm), mean ± SD, range (min. – max.)** | 2.92 ± 0.58 (1.88 – 3.81) | 0.97 ± 0.11 (0.81 – 1.31) | < 0.0001 |

**Table S1:** Comparison of cortical thickness at the ventral and distal patellar cortex. SD = standard deviation, mm = millimetre

| **Parameter (mean ± SD, range [min. – max.])** | **SA (n = 7)** | **TL (n = 7)** | **TA (n = 7)** | **SA vs. TL (P)** | **SA vs. TA (P)** | **TL vs. TA (P)** |
| --- | --- | --- | --- | --- | --- | --- |
| **Bone mineral density (mg/cm³)** | 210.8 ± 51.9 (135.6 – 287.0) | 229.0 ± 31.2 (170.9 – 258.2) | 212.9 ±  51.0 (114.2 – 253.9) | n.s. | n.s. | n.s. |

**Table S2:** Bone mineral density of the different groups and comparison between groups. SD = standard deviation, SA = suture anchor, TL = transosseous longitudinal, TA = transosseous anterior, mm = millimetre, N = Newton, mg/ cm³ = milligram per cubic centimetre

**List of Figures and Tables**

**Figure S1:** Representative computed tomography (CT) images demonstrating cortical thickness measurement sites. (A) Axial image plane: Visualization of ventral cortical bone (highlighted in light blue). Yellow crosses indicate three exemplary measurement points for ventral cortical thickness (lateral, central, medial). (B) Sagittal image plane: Visualization of distal cortical bone at the distal patellar pole (highlighted in light blue). Yellow crosses indicate three exemplary measurement points for distal cortical thickness**.**

**Figure S2:** Boxplot comparing mean cortical thickness (mm) of the ventral patellar region versus the distal patellar pole (apex). **** indicates P < 0.0001. Whiskers represent minimum and maximum values.

**Table S1:** Comparison of cortical thickness at the ventral and distal patellar cortex. SD = standard deviation, mm = millimetre

**Table S2:** Bone mineral density of the different groups and comparison between groups. SD = standard deviation, SA = suture anchor, TL = transosseous longitudinal, TA = transosseous anterior, mm = millimetre, N = Newton, mg/ cm³ = milligram per cubic centimetre

**List of Abbreviations**

BMD:	Bone Mineral Density

CT:	Computed Tomography

HU:	Hounsfield Unit

QCT:	Quantitative Computed Tomography

ROI:	Region of Interest

SA:	Suture Anchor refixation

SD:	Standard Deviation

TA:	Transosseous Anterior refixation

TL:	Transosseous Longitudinal refixation

References

1.	Krappinger D, Roth T, Gschwentner M et al. (2012) Preoperative assessment of the cancellous bone mineral density of the proximal humerus using CT data. Skeletal Radiol 41:299–304. doi: 10.1007/s00256-011-1174-7
